# Comparison of in-hospital complications following acute total hip arthroplasty versus open reduction and internal fixation in patients aged 50 years and older with acetabular fractures

**DOI:** 10.1302/2633-1462.75.BJO-2026-0058.R1

**Published:** 2026-05-13

**Authors:** Geoffrey W. Schemitsch, Graeme Hoit, Matthew Raleigh, Hans J. Kreder, Aaron Nauth, Rob Fowler, Amir Khoshbin

**Affiliations:** 1 Department of Surgery, Division of Orthopaedic Surgery, University of Toronto, Toronto, Canada; 2 Institute of Health Policy, Management and Evaluation, University of Toronto, Toronto, Canada; 3 Division of Orthopaedic Surgery, Sunnybrook Health Sciences Centre, Toronto, Canada; 4 Division of Orthopaedic Surgery, St. Michael’s Hospital, Toronto, Canada; 5 Department of Critical Care Medicine, Sunnybrook Health Sciences Centre and University of Toronto, Toronto, Canada

**Keywords:** Acetabulum fracture, Total hip arthroplasty, Open reduction internal fixation, Complications, Trauma, acetabulum fractures, open reduction and internal fixation (ORIF), acute total hip arthroplasty, trauma, medical complications, propensity score matching, propensity score, postoperative medical complications, retrospective cohort study, Wilcoxon signed-rank tests

## Abstract

**Aims:**

To examine the relationship between surgical treatment type (acute total hip arthroplasty (THA) vs open reduction and internal fixation (ORIF)) and in-hospital medical complications in older adult trauma patients with operatively managed acetabulum fractures.

**Methods:**

We conducted a retrospective cohort study of patients aged ≥ 50 years who presented to institutions participating in the Trauma Quality Improvement Program between 1 January 2017 and 31 December 2022, and who underwent acetabulum fracture surgery within three weeks of admission. Our primary outcome was the development of in-hospital medical complications. Secondary outcomes included each medical complication alone, hospital length of stay, and discharge disposition. Acute THA patients were matched 1:1 without replacement to patients treated with ORIF on the logit of the propensity score using a greedy nearest-neighbour matching algorithm. Generalized estimating equations were used to calculate percent absolute risk differences with 95% CIs for categorical outcomes in the propensity score-matched sample. Wilcoxon signed-rank tests were used compare within pair differences in continuous outcomes.

**Results:**

A total of 10,213 patients were included in our study, of which 1,226 (12%) were treated with an acute THA and 8,987 (88%) were treated with ORIF. A total of 1,223 acute THA patients were matched to 1,223 ORIF patients. After matching, there were no meaningful differences in any baseline characteristics between the two treatment groups. There was no difference in the risk of in-hospital complications between patients treated with acute THA (216/1,223 (17.7%) vs patients who were treated with ORIF (201/1,223 (16.4%)) (absolute risk difference 1.23%, 95% CI -1.71 to 4.17, p = 0.414). There were no significant differences in the risk of each complication, length of stay, or discharge disposition.

**Conclusion:**

Our results suggest that acute THA and ORIF demonstrate similar risks of postoperative medical complications among older patients with acetabular fractures.

Cite this article: *Bone Jt Open* 2026;7(5):643–650.

## Introduction

Acetabulum fractures are complex intra-articular injuries involving the pelvic portion of the hip joint.^1^ For surgical candidates, the most common treatment approach involves open reduction and internal fixation (ORIF) to restore hip joint congruency and minimize the risk of developing post-traumatic hip arthritis.^1-3^ The onset of symptomatic post-traumatic arthritis requiring conversion to a total hip arthroplasty (THA) following ORIF has been estimated to occur in 18% of cases.^4^ Conversion THA procedures can be complicated by the presence of fixation hardware, heterotopic ossification, and fibrous tissue secondary to the index ORIF, and are associated with an elevated risk of infection and dislocation.^5^

Advancements in arthroplasty components have prompted increased interest in acetabulum fracture management with THA in the acute setting.^2,6,7^ A recent study of the Swedish Fracture Register found that 23% of operatively managed acetabulum fractures were treated with an acute THA between 2014 and 2020.^8^ Acute THA eliminates the risk of post-traumatic arthritis, as the injured hip is replaced with a prosthesis. Additionally, acute THA may accelerate postoperative mobilization, which is crucial in elderly patients.^9^ However, acute THA may be associated with greater surgical morbidity when compared with ORIF, as prolonged operating times, high rates of blood loss, and greater surgical complexity have been reported.^10^ It is uncertain whether this translates into differences in perioperative morbidity. To date, most studies have evaluated important implant-related outcomes such as the risk of dislocation,^11,12^ periprosthetic fractures,^12^ or reoperation,^13-16^ while the evidence regarding the perioperative safety of each treatment in elderly trauma patients has predominantly been limited to non-comparative case series or studies with relatively small acute THA samples.^17,18^

The aim of our study was to examine the relationship between treatment type (THA vs ORIF) and in-hospital medical complications in a sample of adult patients aged over 50 years with operatively managed acetabulum fractures presenting to trauma programmes in North America. We hypothesized that there would be no association between treatment type and the development of in-hospital complications.

## Methods

### Data source and setting

This investigation was a retrospective cohort study using administrative data from the American College of Surgeons Trauma Quality Improvement Program (TQIP) registry.^19^ TQIP collects data from patients with severe injuries (abbreviated injury score ≥ 3 in at least one body region) presenting to over 750 participating trauma centres across the USA and Canada.^19^ TQIP tracks outcomes that occur during trauma-related admissions such as mortality, in-hospital complications, length of stay, and discharge status.^20^ Data abstraction is completed by trained individuals and quality assurance checks are completed through external inter-rater reliability audits.^19^ This project was approved by the Unity Health research ethics board (Toronto, Canada). The need for patient-informed consent was waived due to the deidentified nature of the data. This study was reported according to the REporting of studies Conducted using Observational Routinely-collected health Data (RECORD) statement (Supplementary Material).^21^

### Patient characteristics

At baseline, patients treated with acute THA were older (mean age 67.7 years (SD 9.9) vs 62.1 years (SD 9), standardized difference = 0.6); more likely to be female (40.4% vs 27.1%, standardized difference = 0.28); less likely to be insured by Medicaid (5% vs 10.6%, standardized difference = 0.21); and more likely to identify as White race (85.3% vs 77.4%, standardized difference = 0.2) and non-Hispanic ethnicity (96.2% vs 91.2%, standardized difference = 0.2) (Table I).

**Table I.** Baseline characteristics of cohort before and after propensity score matching.

Characteristic	Before matching			After matching		
	THA (n = 1,226)	ORIF (n = 8,987)	SMD	THA (n = 1,223)	ORIF (n = 1,223)	SMD
**Demographic characteristics, n (%) unless otherwise specified**						
Mean age, yrs (SD)	67.7 (9.9)	62.1 (9)	0.6	66.8 (10.2)	67.7 (9.9)	0.01
Female	495 (40.4)	2437 (27.1)	0.28	493 (40.3)	483 (39.5)	0.02
Non-White race	180 (14.7)	2029 (22.6)	0.2	180 (14.7)	163 (13.3)	0.04
Hispanic ethnicity	47 (3.8)	790 (8.8)	0.2	47 (3.8)	56 (4.6)	0.04
**Insurance status**						
Private insurance	533 (43.5)	4496 (50)	0.13	532 (43.5)	522 (42.7)	0.02
Medicare	508 (41.4)	2282 (25.4)	0.34	506 (41.4)	510 (41.7)	0.01
Medicaid	61 (5)	948 (10.6)	0.21	61 (5)	66 (5.4)	0.02
Self-pay	43 (3.5)	646 (7.2)	0.16	43 (3.5)	39 (3.2)	0.02
Other	81 (6.6)	615 (6.8)	0.01	81 (6.6)	86 (7)	0.02
**Comorbidities, n (%) unless otherwise specified**						
Alcohol use disorder	66 (5.4)	682 (7.6)	0.09	66 (5.4)	77 (6.3)	0.04
Angina	2 (0.2)	17 (0.2)	0.01	2 (0.2)	0 (0)	0.01
Anticoagulant use	174 (14.2)	668 (7.4)	0.22	172 (14.1)	174 (14.2)	< 0.01
CHF	64 (5.2)	276 (3.1)	0.11	63 (5.2)	67 (5.5)	0.01
Cirrhosis	10 (0.8)	109 (1.2)	0.04	10 (0.8)	13 (1.1)	0.03
COPD	155 (12.6)	657 (7.3)	0.18	153 (12.5)	166 (13.6)	0.03
CVA	34 (2.8)	144 (1.6)	0.08	34 (2.8)	38 (3.1)	0.02
Dependent functional status	116 (9.5)	305 (3.4)	0.25	115 (9.4)	109 (8.9)	0.02
Dementia	58 (4.7)	130 (1.5)	0.19	57 (4.7)	55 (4.5)	0.01
Diabetes	277 (22.6)	1799 (20)	0.06	276 (22.6)	261 (21.3)	0.03
Disseminated cancer	11 (0.9)	36 (0.4)	0.06	11 (0.9)	13 (1.1)	0.02
Hypertension	686 (56)	3946 (43.9)	0.24	684 (55.9)	685 (56)	< 0.01
Myocardial infarction	10 (0.8)	70 (0.8)	< 0.01	10 (0.8)	16 (1.3)	0.05
Peripheral arterial disease	19 (1.6)	79 (0.9)	0.06	19 (1.6)	18 (1.5)	0.01
Mental health disorder	133 (10.9)	934 (10.4)	0.01	133 (109)	139 (11.4)	0.01
Renal failure	29 (2.4)	89 (1)	0.11	28 (2.3)	25 (2)	0.02
Smoking	245 (20)	2147 (23.9)	0.09	245 (20)	250 (20.4)	0.01
Substance misuse	59 (4.8)	679 (7.6)	0.11	59 (4.8)	62 (5.1)	0.01
Mean TCI (SD)	0.11 (0.5)	-0.02 (0.5)	0.27	0.11 (0.5)	0.11 (0.5)	0.01
**Injury characteristics, n (%) unless otherwise specified**						
GCS ≤ 8	43 (3.5)	659 (7.3)	0.17	43 (3.5)	35 (2.9)	0.02
Mean SBP, mmHg (SD)	134.3 (28.6)	127.0 (28.7)	0.25	134.2 (28.6)	133.9 (27.3)	0.01
Mean heart rate, BMP (SD)	88.2 (18.6)	91.1 (20.2)	0.15	88.2 (18.7)	88 (18.3)	0.01
ISS 9 to 16	673 (54.9)	3129 (34.8)	0.41	670 (54.8)	674 (55.1)	0.01
ISS 17 to 25	376 (30.7)	3566 (39.7)	0.19	376 (30.7)	380 (31.1)	0.01
ISS ≥ 26	177 (14.4)	2292 (25.5)	0.28	177 (14.5)	169 (13.8)	0.02
Blood transfusion within 4 hrs	145 (11.8)	2172 (24.2)	0.33	145 (11.9)	154 (12.6)	0.02
Embolization procedure	31 (2.5)	591 (6.6)	0.2	31 (2.5)	32 (2.6)	0.01
**Post-ED destination, n (%)**						
Regular ward	504 (41.1)	2272 (25.3)	0.34	501 (41)	488 (39.9)	0.02
Step-down/observation unit	123 (10)	860 (9.6)	0.02	123 (10.1)	118 (9.7)	0.01
Intensive care unit	428 (34.9)	4233 (47.1)	0.25	428 (35)	436 (35.7)	0.01
Operating theatre	171 (14)	1620 (18)	0.11	171 (14)	181 (14.8)	0.02
**Facility characteristics, n (%)**						
Wait time > 72 hrs	468 (38.2)	3510 (39.1)	0.02	466 (38.1)	470 (38.4)	0.01
Teaching status	688 (56.1)	5248 (58.4)	0.05	688 (56.3)	699 (57.2)	0.02
Level I trauma centre	808 (65.9)	6118 (68.1)	0.05	808 (66.1)	824 (67.4)	0.03
**Number of hospital beds (%)**						
< 200	260 (21.2)	1842 (20.5)	0.02	259 (21.2)	272 (22.2)	0.03
201 to 400	206 (16.8)	1486 (16.5)	0.01	206 (16.8)	205 (16.8)	0.02
401 to 600	297 (24.2)	2402 (26.7)	0.06	297 (24.3)	294 (24)	0.01
> 600	463 (37.8)	3257 (36.2)	0.03	461 (37.7)	452 (37)	0.02

BMP, beats per minute; CHF, congestive heart failure; COPD, chronic obstructive pulmonary disorder; CVA, cerebrovascular accident; GCS, Glasgow Coma Scale; ISS, Injury Severity Score; ORIF, open reduction and internal fixation; SBP, systolic blood pressure; SMD, standardized mean difference; TCI, Trauma Comorbidity Index; THA, total hip arthroplasty.

### Inclusion and exclusion criteria

We included patients aged ≥ 50 years who presented to institutions participating in TQIP between 1 January 2017 and 31 December 2022, with acetabulum fractures which were treated surgically with either acute THA or ORIF. Acetabulum fracture diagnosis was confirmed using International Classification of Diseases of the World Health Organization, tenth revision (ICD-10) diagnostic codes. Operative treatment with either ORIF or an acute THA procedure (THA alone or combined hip procedure, whereby a THA and ORIF are performed concurrently) was classified using ICD-10 procedure codes (Supplementary Material).^17^ Patients with a definitive treatment date occurring beyond three weeks of hospital admission were excluded in keeping with previous time-based definitions of acute THA.^22^ Patients with missing data for variables of interest were also excluded. Missingness was less than 3.3% for all included variables.

### Exposure and outcomes

The primary exposure was the type of surgical management: acute THA compared with ORIF as a control.

Our primary outcome was the development of any in-hospital medical complication. A binary outcome (complication or no complication) was defined as the presence of any of: acute respiratory distress syndrome, ventilator-associated pneumonia, acute kidney injury, pressure injury, unplanned intensive care unit (ICU) admission, deep vein thrombosis, pulmonary embolism, myocardial infarction, sepsis, stroke, cardiac arrest, and/or death. Secondarily, we investigated whether there was any difference in the development of each medical complication alone, discharge disposition, or total hospital length of stay in days.

### Covariates

Patient, injury, and facility characteristics thought to potentially influence the relationship between the type of surgical treatment and medical complications were recorded. Specifically, we evaluated age, sex, race, ethnicity, insurance status, medical comorbidity burden, and substance use as patient-level covariates. Measured medical comorbidities included a history of congestive heart failure, myocardial infarction, angina, hypertension, cirrhosis, chronic obstructive pulmonary disease, cerebrovascular accident, peripheral arterial disease, renal failure, diabetes, disseminated cancer, dementia, psychiatric illness, dependent baseline functional status, and the pre-injury use of anticoagulation medications. Substance use was quantified by a history of alcohol misuse, illicit drug use, or a history of smoking.

Multiple markers of injury severity were identified. We measured Injury Severity Score,^23^ Glasgow Coma Scale score on arrival, vital signs (systolic blood pressure and heart rate) on arrival, post-emergency department destination (regular floor, high observation, ICU, or operating theatre), requirement of a blood transfusion within four hours of hospital presentation, and need for an embolization procedure.

Facility characteristics included hospital bed size, teaching status, trauma level designation, and wait time to definitive fracture surgery.

### Statistical analysis

Descriptive statistics were calculated with means (SD) or median (IQR) as appropriate. Counts and proportions were calculated for categorical variables. The propensity score, for a patient to receive treatment with acute THA, was calculated using a multivariable logistic regression model. Covariates included in the model were selected based on the consensus expert opinion of three trauma fellowship-trained orthopaedic surgeons (AK, AN, HJK), two of whom are trauma/arthroplasty dual fellowship-trained, all with substantial experience in acetabular fixation and/or THA in a trauma setting. Included variables were either thought to be true confounding variables or prognostically associated with the outcome.^24,25^ Instrumental variables were not included in the model.^24^ Acute THA and ORIF patients were matched 1:1 without replacement on the logit of the propensity score using a greedy nearest-neighbour matching algorithm and a caliper width equal to 0.2 of the SD of the logit of the propensity score.^26^ Covariate balance was assessed in the unmatched and matched samples using standardized differences. A threshold standardized difference of 0.1 was used to signify the presence of meaningful covariate imbalance between treatment groups.^27,28^ Differences in our primary and secondary outcomes were compared between the two groups after matching. We used generalized estimating equations to calculate the percent absolute risk difference with 95% CIs between treatment groups for categorical outcomes while accounting for propensity score-matched pairs.^29^ We used the Wilcoxon signed-rank test to compare within pair differences in length of stay in days. All statistical tests were two-sided, assuming a type I error of 0.05. Analyses were conducted using SAS v. 9.4 (SAS Institute, USA). We conducted a complete case analysis assuming data was missing completely at random.

## Results

**Fig. 1:**
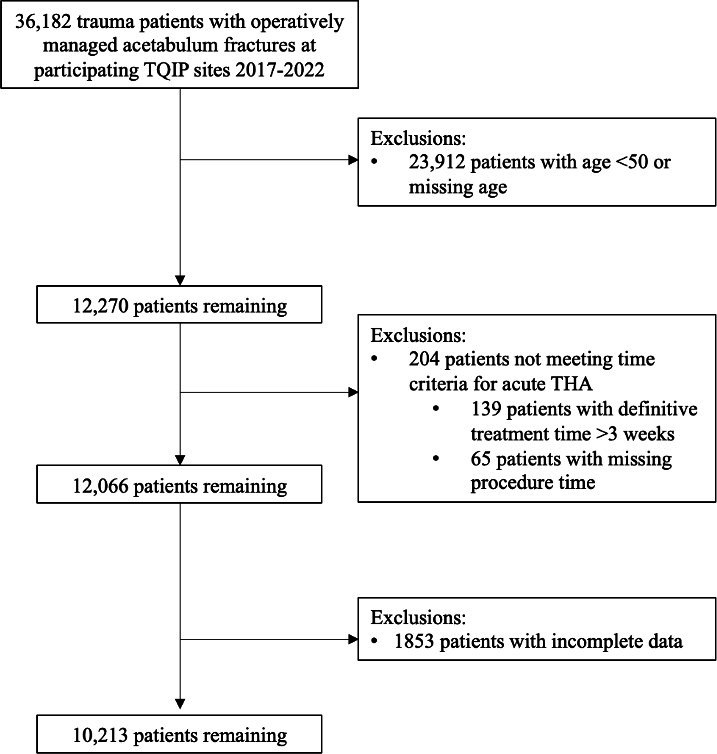
Summary of cohort creation. THA, total hip arthroplasty; TQIP, Trauma Quality Improvement Program.

Of the 36,182 patients who underwent operative management of an acetabulum fracture in the TQIP database, 10,213 were eligible for our propensity score model (Figure 1). Among eligible patients, 1,226 (12%) were treated with an acute THA and 8,987 (88%) were treated with ORIF (Table I). Of the patients treated with acute THA, 617 (50.3%) were treated with acute THA alone and 609 (49.7%) were treated with a combined hip procedure. Acute THA patients demonstrated greater comorbidity burden, as shown by more frequent histories of congestive heart failure, chronic obstructive pulmonary disease, dependent functional status, dementia, hypertension, renal failure, and baseline anticoagulation medication usage. Patients treated with ORIF were more likely to present with higher Injury Severity Scores and Glasgow Coma Scale scores ≤ 8. Overall, patients treated with ORIF demonstrated evidence of higher acuity trauma as shown by higher rates of intensive care unit admission, immediate operating theatre disposition, blood transfusions within four hours, and embolization procedures for haemorrhage control.

A total of 1,223 acute THA patients (99.8%) were each matched to a patient who was treated with ORIF. After propensity score matching, there were no meaningful differences in any of the measured baseline characteristics between the two treatment groups (Table I). Of the 1,223 matched patients treated with acute THA, 216 (17.7%) experienced a medical complication compared with 201 patients (16.4%) of the 1,223 who were treated with ORIF (absolute risk difference 1.23%, 95% CI -1.71 to 4.17, p = 0.414). There were no significant differences in the occurrence of each specific medical complication or discharge disposition between the two groups (Table II). There was no significant difference in hospital length of stay (within-pair median difference 0 days (IQR -6 to 5), p = 0.224).

**Table II.** Primary and secondary outcomes after propensity score matching.

Outcome	Number of patients (%)		Absolute risk difference, % (95% CI)	p-value
	THA (n = 1,223)	ORIF (n = 1,223)		
**Primary outcome**				
Any medical complication	216 (17.7)	201 (16.4)	1.23 (-1.71 to 4.17)	0.414
**Secondary outcomes, n (%)**				
Death	38 (3.1)	50 (4.1)	-0.98 (-2.47 to 0.5)	0.195
Cardiac arrest	13 (1.1)	19 (1.6)	-0.49 (-1.40 to 0.42)	0.289
Myocardial infarction	6 (0.5)	4 (0.3)	0.16 (-0.34 to 0.67)	0.527
Stroke	14 (1.1)	9 (0.7)	0.41 (-0.36 to 1.18)	0.297
Deep vein thrombosis	50 (4.1)	35 (2.9)	1.23 (-0.23 to 2.68)	0.099
Pulmonary embolism	19 (1.6)	23 (1.9)	-0.33 (-1.34 to 0.69)	0.527
Acute respiratory distress syndrome	11 (0.9)	11 (0.9)	0 (-0.75 to 0.75)	1.00
Ventilator associated pneumonia	14 (1.1)	13 (1.1)	0.08 (-0.75 to 0.91)	0.847
Sepsis	13 (1.1)	16 (1.3)	-0.25 (-1.11 to 0.62)	0.577
Acute kidney injury	35 (2.9)	31 (2.5)	0.33 (-0.89 to 1.55)	0.599
Unplanned ICU admission	81 (6.6)	62 (5.1)	1.55 (-0.29 to 3.4)	0.099
Pressure injury	33 (2.7)	23 (1.9)	0.82 (-0.38 to 2.02)	0.181
Discharge disposition				
Home	263 (21.5)	265 (21.7)	-0.16 (-3.36 to 3.03)	0.920
Rehabilitation facility	132 (10.8)	115 (9.4)	1.39 (-0.97 to 3.75)	0.248
Secondary care hospital*	355 (29)	380 (31.1)	-2.04 (-5.64 to 1.56)	0.266
Skilled nursing facility	435 (35.6)	413 (33.8)	1.8 (-1.92 to 5.51)	0.343
Deceased	38 (3.1)	50 (4.1)	-0.98 (-2.47 to 0.5)	0.195
**Continuous outcomes**			Within-pair difference	
Median hospital length of stay, days (IQR)	11 (7 to 16)	11 (8 to 16)	0 (-6 to 5)	0.224

* Secondary care hospital refers to discharge/transfer to a short-term general hospital, long-term care hospital, or psychiatric hospital/unit.

ICU, intensive care unit; ORIF, open reduction internal fixation; THA, total hip arthroplasty.

## Discussion

In this multicentred retrospective cohort study, we found no difference in our primary outcome – the occurrence of in-hospital medical complications – in older patients with acetabulum fractures who were managed with either acute THA or ORIF. We also found no difference in secondary outcomes, including hospital length of stay or discharge disposition, between the two groups. These findings suggest that treatment with acute THA is as safe as ORIF alone in the older adult trauma patient population during the index hospital admission.

Previous literature evaluating the perioperative safety of acute acetabulum fracture management with THA is sparse. In a retrospective case series, Kelly et al^17^ reported that 129 of 956 patients (13.2%) who were treated with acute THA developed a perioperative medical complication. The most common medical complications in their investigation were renal failure (9.1%), respiratory failure (4.4%), and sepsis (4.1%).^17^ In comparison, medical complications following acute THA were more common in our study, occurring in 17.7% of the matched sample, which may be attributable to the higher injury severity captured by the TQIP database. Denyer et al^18^ evaluated the safety of treatment with combined acute THA with ORIF versus ORIF alone in a sample of 1,187 patients with acetabulum fractures. In unadjusted analyses of patients with associated fracture patterns, the authors found significantly higher rates of pneumonia and postoperative transfusions in the acute THA group as well as non-significantly higher rates of death (10% vs 3.2%). In a multivariable logistic regression stratified by fracture type, the authors found no difference in complications between the two groups.^18^ In contrast to our findings, the authors demonstrated that treatment with an acute THA significantly reduced total hospital length of stay. The study only captured 184 acute THA procedures, which limited their ability to control for potentially important confounding variables.^18^

Our study has notable strengths. There is substantial practice variation in the management of acetabular fractures in older patients, with no clear guidelines or consensus statements available to date.^30^ Nevertheless, the treatment of acetabulum fractures with acute THA has grown substantially in recent years.^8,31^ We used administrative data from the TQIP database, which draws from a large volume of trauma centres in North America. This included patients who presented with fractures from 2017 to 2022, which captures the evolving practice patterns in this patient population. Moreover, restricting our analysis to patients aged > 50 years created a study sample that was reflective of the typical age group for whom providers may begin to consider treatment with acute THA, as the trade-off between future revision arthroplasty and the development of post-traumatic hip arthritis becomes clinically relevant.^32^ Lastly, our variable selection was based on the consensus expert opinion of one trauma fellowship-trained and two trauma/arthroplasty dual-trained orthopaedic surgeons, to assist with proper specification of our propensity score model. We included several patient-, injury-, and facility-level covariates that may influence the development of postoperative medical complications in patients with acetabulum fractures. Before propensity score matching, we observed several meaningful differences in baseline covariates between treatment groups. After matching, no between-group differences remained among measured variables.

Although we accounted for several patient, injury, and facility level covariates, it is possible that our findings could be influenced by residual unmeasured confounding or modelling deficiencies. We were unable to account for different acetabulum fracture types in our analyses. Certain fracture types, such as associated both column and transverse posterior wall patterns, may require greater surgical exposure and instrumentation, which may lead to different risks of perioperative medical complications. Secondly, our outcome of interest was the occurrence of in-hospital perioperative medical complications. Certain fracture-related characteristics such as posterior wall comminution, marginal impaction, femoral head impaction, and associated fracture types have demonstrated associations with failed ORIF leading to salvage THA.^4,33,34^ In our study, any complications developed following discharge from hospital were not captured. Therefore, we cannot comment on the occurrence of important surgical outcomes such as dislocation, periprosthetic fracture, implant-related infection, and revision surgery. Previous studies have suggested a reduction in the risk of reoperation for elderly patients treated with acute THA when compared with those treated with ORIF.^35,36^ This represents an area of ongoing investigation. Finally, while the TQIP database uses rigorous data quality checks, our investigation may be limited by potential biases or errors inherent to the use of administrative health data such as misclassification of covariates and outcomes.

In conclusion, our study demonstrated that acetabulum fracture management with acute THA was associated with similar rates of in-hospital medical complications when compared with ORIF in a sample of adult trauma patients aged 50 years and older. Future research should evaluate the relative treatment efficacy of acute THA and ORIF, specifically comparing patient-reported outcomes, reoperation rates, and the occurrence of implant-related complications over extended periods of follow-up.

**Take home message**

- Acute management with total hip arthroplasty for acetabular fracture in adults aged ≥ 50 years is associated with similar risks of perioperative medical complications when compared to propensity score matched patients treated with open reduction and internal fixation.

- There was also no difference in discharge disposition or length of stay between treatment groups.

## Data Availability

The datasets generated and analyzed in the current study are not publicly available due to data protection regulations. Access to data is limited to the researchers who have obtained permission for data processing. Further inquiries can be made to the corresponding author.
